# Drug Delivery of Solid Lipid Nanoparticles (SLNs) and Nanostructured Lipid Carriers (NLCs) to Target Brain Tumors

**DOI:** 10.34172/apb.2023.062

**Published:** 2022-11-05

**Authors:** Soheil Mehrdadi

**Affiliations:** Department of Pharmaceutical and Pharmacological Sciences, University of Padova, Padua, Italy

**Keywords:** Brain tumors, Glioblastoma multiforme, Blood-brain barrier, Solid lipid nanoparticles, Nanostructured lipid carriers, Brain drug delivery

## Abstract

Brain, predisposed to local and metastasized tumors, has always been the focus of oncological studies. Glioblastoma multiforme (GBM), the most common invasive primary tumor of the brain, is responsible for 4% of all cancer-related deaths worldwide. Despite novel technologies, the average survival rate is 2 years. Physiological barriers such as blood-brain barrier (BBB) prevent drug molecules penetration into brain. Most of the pharmaceuticals present in the market cannot infiltrate BBB to have their maximum efficacy and this in turn imposes a major challenge. This mini review discusses GBM and physiological and biological barriers for anticancer drug delivery, challenges for drug delivery across BBB, drug delivery strategies focusing on SLNs and NLCs and their medical applications in on-going clinical trials. Numerous nanomedicines with various characteristics have been introduced in the last decades to overcome the delivery challenge. Solid lipid nanoparticles (SLNs) and nanostructured lipid carriers (NLCs) were introduced as oral drug delivery nanomedicines which can be encapsulated by both hydrophilic and lipophilic pharmaceutical compounds. Their biocompatibility, biodegradability, lower toxicity and side effects, enhanced bioavailability, solubility and permeability, prolonged half-life and stability and finally tissue-targeted drug delivery makes them unique among all.

## Introduction

**Figure 1:**
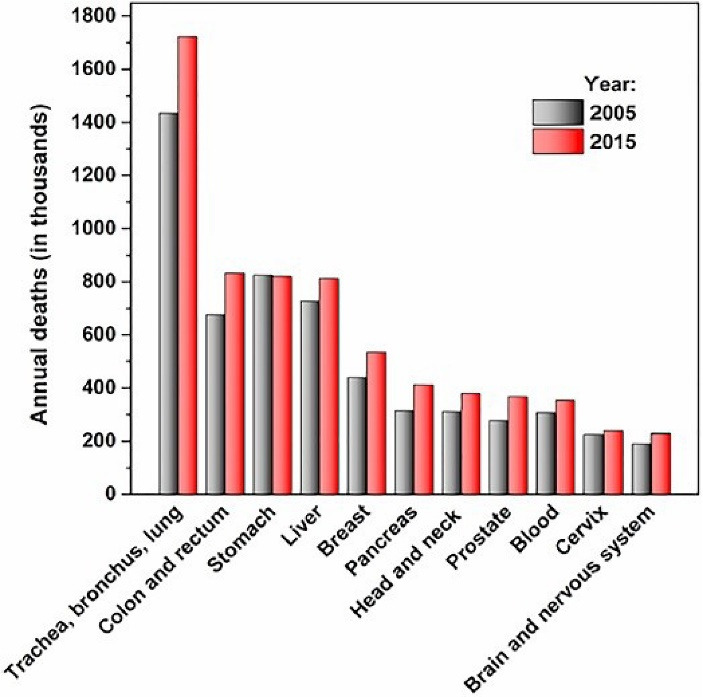
Cancer world demography (2005-2015)

 Cancers, the second cause of global mortality, are accounted for seven to nine million deaths from 2005 to 2015 (Figure 1).^1^ Despite significant progress in diagnosis, treatment and prevention studies of cancer in the last decades, the mortality rate of most cancer cases, especially brain tumors, is still considerable and remains a medical challenge.

 Glioblastoma multiforme (GBM), the most invasive and common malignant tumor of brain (55% of all cases),^2^ has two types: primary (90%) and secondary (10%): the former develops acutely with no previous lower grade pathological signs or symptoms, while the latter which is seen mostly at younger ages arises from evolving and transforming astrocytomas. Both have different genetic profiles and are detectable by specific cell markers, although clinically similar.^3^ Surgical excision of the tumor, co-temporaneous post-surgical radiotherapy and chemotherapy are the therapeutic regimen. Nevertheless, the survival rate is still 1 to 2 years (3–5%).^4^

 The abovementioned therapeutic regimen does not offer a practical solution to target brain tumors in an effective way which can be justified due to the failure of anticancer drugs to exert their maximum effect mainly due to the following: (a) overexpression of P-glycoprotein (P-gp) receptors in GBM chemoresistant cancer cells causing increased drug efflux; (b) hypoxic tumor tissues further aggravating drug delivery by promoting ischemia; (c) high heterogeneity and variability of GBM at molecular, histopathological and genetic levels^5,6^; (d) the effect of treatment on cancer tissue is unpredictable^7^; and (e) the intrinsic feature of GBM; sustaining proliferative signaling, evading growth suppressors, activating invasion and metastasis, enabling replicative immortality, inducing angiogenesis, resisting cell death, evading immune destruction and reprogramming cellular energetics. The highly-resistant character of GBM cancer cells makes them relapse and penetrate healthy brain tissues quickly resulting from their unique cellular heterogeneity, presenting a challenging case in cancer patient management.

 Searching for a brain tumor targeted drug delivery system, numerous “nanomedicines” and de-novo chemotherapeutics have been investigated to overcome low drug penetration and cellular resistance,^4^ and to specifically and accurately deliver the drug to their target tissue without having adverse effects on the adjacent intact tissues.^8^ Despite promising in-vitro results, most in-vivo studies fail when administered systemically mainly due to the biological and pathological barriers, namely as blood-brain barrier (BBB), blood-brain tumor barrier (BBTB), arachnoid barrier and blood cerebrospinal fluid barrier (BCSF) barrier, which restrict the delivery of chemotherapeutic drugs to the tumor tissue.^9^ Hence, it is necessary to investigate these more profoundly.

 The focus of the present mini review is on two types of drug delivery systems, solid lipid nanoparticles (SLNs) and nanostructured lipid carriers (NLCs), to target brain tumors. But, it is worthwhile to discuss first the barriers for brain anticancer drug delivery.

## Barriers to brain drug delivery

###  Blood-brain barrier

 BBB by modulating the transfer of different substances from the blood to CNS maintains a stable concentration of endogenous and exogenous substances in the body which are selected according to their structural and molecular features.^10^ BBB originates from the neurovascular unit which is extended along the cerebral capillaries, and is formed by endothelial cells, pericytes, vascular smooth muscle cells, neurons, astrocytes and perivascular macrophages.^11^ However, there are anatomical differences between brain and peripheral capillaries; the peripheral capillaries lack fenestrations and intercellular pores (while present in brain capillaries) because of endothelial tight and adherent junctions which limit transportation through paracellular pathways. Another difference is related to their components; tight junctions are composed of cingulin, occluding, claudins and zonula occludens proteins, while adherens junctions are cadherins.^11^ Different molecules (e.g. nutrients, enzymes and proteins) are transferred inside and outside of the CNS mediated by four mechanisms (Table 1). The unique structure of BBB restricts the penetration of almost 98% of new therapeutic molecules into the brain tissue following systemic administration which poses a serious problem to access the brain microvasculature (20 m^2^ surface area, 640 km length).^12^ This large and long vascularization provides a rapid transportation (by simple diffusion) of tiny molecules less than a second,^13^ which is almost impermeable through the peripheral circulation.^13^ Furthermore, periendothelial accessory structures (astrocytes) act to mediate and produce growth factors and cytokines, keep potassium ion levels and inactivate neurotransmitters.^14^

**Table 1:** Different mechanisms of physiological transport across BBB

**Pathway**	**Transporter **	**Process**	**Feature of molecules**	**Note**	**Reference**
Paracellular diffusion	Across cells	Non-saturable, non-competitive	Small water-soluble	Limited diffusion due to endothelial cells tight junction	^15^
Transcellular diffusion	Across cells	Non-saturable, non-competitive	High lipophilicity, unfit for active efflux transport, low molecular weight ( < 400 Dalton)	-	^15,16^
Carrier- mediated transport	Glucose transporters (GLUT1 & GLUT3), monocarboxylate (lactate; pyruvate) transporter system (MCT1), specialized carriers (essential amino acids and vitamins)	Active transport by selective membrane- bound carrier systems	Glucose, galactose, amino acids, nucleosides, lactates and pyruvates, adenine and guanine, choline, vitamins and hormones	Establishment of transient narrow pores	^15,17-19^
Receptor- mediated endocytosis	Specific receptors on the luminal side of the barrier for insulin, insulin-like growth factors, angiotensin II, folates and transferrin	Absorptive transcytosis without specific plasma- membrane receptors	Endogenous proteins and hormones, low- density lipoproteins (LDL), Polycationic proteins (albumins and immunoglobulins)	Polycationic substances bound to negative charges of endothelial cells of plasma membrane	^15,20,21^

 The different “drug delivery” transport mechanisms across BBB have the principal role of delivering drugs, considering that pro-drugs and drugs, resembling endogenous compounds and nutrients, are encapsulated according to the required therapeutic dosage of compounds and nutrients.^13^

 Besides, it is proved that the P-gp receptors (an ATP-dependent drug transport protein) on the BBB endothelial cells interfere with the penetration of molecules inside by exporting them outside the cells. Overcoming P-gp drug export mechanism could maximize massively the drug absorption into the brain tissue.^22,23^

## Blood-brain tumor barrier

 In definition, when a brain tumor is formed, the BBB will be based between this new tumor tissue and capillary vessels. Thus, the BBB is referred to as the BBTB.^24^ According to the shape and permeability of the BBTB, there are three stages which mainly rely on the progression of adjacent brain tumor changes.^24^

 In stage one (early-stage malignant tumor), the BBTB capillaries are consistent, non-fenestrated and intact, consequently the normal brain capillaries are still being supplied with necessary growth nutrients.^25^ However, during stage two which is tumor growth, after the adjacent intact brain tissues are invaded by cancer cells and the tumor tissue enlarges more than 2 mm, new capillaries form by the angiogenesis mechanism (neovasculature) which are consistent with fenestrations of about 12 nm. This in turn changes the permeability and subsequently only molecules smaller than twelve nm can infiltrate inside BBTB.^25,26^ In stage three, inter-endothelial gaps are established between cerebral endothelial cells (CECs) due to continuous tumor growth which consequently damage the BBTB integrity.

 A study in mouse reported thinner than normal microvessel basement membrane of CECs with 1 μm inter-endothelial gaps and 48 nm fenestration size.^25^ The high permeability of BBTB can be used to accumulate higher doses of anticancer drugs in the tumor site.^27^

 The damaged BBTB forms tiny holes to compensate for their high metabolic demands and this phenomenon is widely seen in GBM. Hypoxia triggers the angiogenesis in particular zones of GBM and ultimately damages the BBB integrity.^28^ Although, GBM spread aggressively and rapidly in the adjacent intact tissues this does not affect the BBTB with enhanced permeability and retention. Therefore, overcoming the BBTB promotes higher anticancer drug delivery to GBM tumors.^29,30^

## Tumor microenvironment

 In order to have a more efficient drug delivery and successful treatment of brain cancers, a complete studying of tumor microenvironment (TME) is also necessary to interpret and justify the tumor molecular and biological processes.^31,32^ Generally, TME is considered as a complex heterogeneous environment where different physiological factors, such as mechanical stresses and protein-binding drug degradation, is required to be evaluated for a drug delivery system. So far, various components of TME have been studied and explained, namely as extracellular matrix (microglia, astrocytes and neurons) and blood vessels (also the ones forming the BBB), through the latter the vital role of immune and lymphatic systems and vascular cells in successful brain tumor treatment has been proved.^33-38^

 Macrophages of the myeloid cells and microglia – collectively referred to as tumor-associated macrophages (TAMs)^39,40^ – have been provoked by GBM cells to generate immunosuppressive tumor- related cytokines and elevated T-cell apoptosis.^41^ Moreover, GBM cells also inhibit antitumor immune system activities by preventing immune-stimulatory cytokines’ production and activating the regulatory T-cells.^42^ Further studies suggest that preventing TAMs activity and readjusting them phenotypically to be functional against tumor progression is more efficacious than deactivating them,^32,43^ and this concept has been the basis of the innovation of the recently introduced anti-cancer therapies; immune-checkpoint inhibitors, cellular therapies (such as chimeric antigen receptor T cells) and vaccines.^42-44^

 Nevertheless, unlike their promising features as a new approach in cancer therapy,^31^ there are factors hampering their maximum therapeutic effect in tumor tissue, the most important one is the BBB and its contribution toward immunotherapies penetration and other chemotherapeutics (such as Temozolomide) local co-existence. It is commonly believed that tumor vessels are significantly more permeable than the ones in intact tissues,^45^ and could diffuse more blood from vessels into the interstitial space to increase the interstitial fluid pressure.

 The tumor vessels lose their well-established structure with the progression of tumor; they are not as functional as arteries, capillaries and veins in intact tissues. Decreased blood velocity and pressure gradient,^46,47^ increased interstitial fluid pressure and suppressed transvascular convection prevent drug delivery to the cancer tissue and subsequently limits the drug diffusion.^48,49^ Furthermore, the systemic administration diminishes even more delivery of chemotherapeutic by exposing them to enzymatic/hydrolytic degradation. Hence, only small doses reach the cancer site with hypoxia and low pH which further aggravate the situation and the administered drug will not exert a therapeutic effect.

## Challenges for oral delivery of anticancer drugs across BBB

 The oral route to target brain cancers is a striking challenge, in part due to the limited oral absorption, short plasma half-life and the BBB as a barrier. The oral delivery of hydrophilic drugs to brain requires absorption and infiltration through the gastrointestinal tract (GIT) and then across the BBB. Moreover, the hydrogen bonds formed between hydrophilic drugs and GIT aqueous contents, hindering the absorption, further limits epithelial infiltration process.^50^ Additionally, peptide drugs are rapidly degraded upon their administration in GIT and this decreases their plasma half-lives.^51^

## The application of nanomedicines for brain anticancer drug delivery

 During recent decades, various nanomedicines of different size, material (Synthetic, natural, organic and inorganic) and shape have been employed for cancer treatment,^52,53^ and these components of construction affect their characteristics. Since their early introduction they have been rapidly evolved, mostly due to replacing unfit conventional clinical techniques with improved ones for refractory diseases, and since then have found their application in pharmaceutical and biomedical industries.^54^

 In order to exert their maximum therapeutic effect with the lowest side effects, effective nanomedicines must be precisely engineered to target the complex pathophysiology of each disease. Therefore, they could be modified in such a way to be responsive according to different endogenous or exogenous stimuli; this feature can be employed for controlled and sustained release of loaded drug. Besides their application for therapeutic purposes, some of them (e.g. inorganic and polymeric/lipid nanoparticles) have been employed for diagnosis purposes. Their dual-purpose application both as therapeutic and diagnostic resulted in the introduction of “theranostic” nanoparticles for which, among all, only synthetic polymers are used.

 Each disease possesses its own specific etiology which is also influenced highly by environmental and genetic factors, and all these differences apply a very specific drug delivery design for targeting the affected area with minimum adverse effect on adjacent intact tissues resulting in tumor regression or complete healing. With regard to the CNS diseases, they all share one challenge: drug delivery through the BBB. In order to overcome this issue, a huge range of therapeutics such as drugs (functionalized with specific targeting segments), DNA/RNA, genes, enzymes, antioxidants can be precisely loaded into the matrix of polymeric/lipid nanomedicines. In some studies on brain cancer, the majority of synthetic nanomedicines used are polymers such as polyethylene glycol (PEG) or Poly(lactide-co-glycolide) with different surface/physicochemical properties, size and shape.^55,56^ however, their degradation produces acidic by-products which are toxic for brain tissue, making them unsuitable for extended drug delivery application, hence, inorganic nanoparticles (still toxic) and lipid-based structures have been introduced.

 With regard to brain cancers, drug delivery happens at local or systemic level; the former will permeate the BBB, accumulating a higher concentration of drug with minimum adverse effects for adjacent intact tissues, evading also degradation/hydrolysis and clearance processes before its in-site delivery. Implants, intraventricular/intrathecal and convection enhanced delivery (CED) all can be named as examples of local drug delivery. The latter, systemic drug delivery on the other hand, is the most common one in terms of compliance for patients since it doesn’t require surgical or clinical intervention, with possibility of repeated doses which makes them less invasive and more favorable compared to the local drug delivery.

 So far, numerous nanomedicines with various size, surface charge and hydration and targeting character have been exploited for brain anticancer delivery, among them nanoparticles, nanofibers and hydrogels (Polymer-based nanostructures), lipid nanocapsules, liposomes, SLNs and NLCs (lipid-based nanostructures) have been widely studied.

 SLNs and NLCs belong to lipid-based nanosystems of drug delivery. They offer better advantages over the polymeric or inorganic nanoparticles; biocompatibility, better penetration through the BBB without any structural modification influencing their function and easy large-scale production. Nevertheless, their low loading capacity is a disadvantage for their clinical application hence the low number of systems marketed commercially.^57^

## Solid lipid nanoparticles

 Seeking for a substitute nanostructured system with lower toxicity, higher loading capacity and stability SLNs were developed and introduced thirty years ago,^58^ and since then numerous studies have proved their efficacy and advantage over emulsions, micelles, polymeric nanoparticles and liposomes.^59^

 SLNs are composed of different lipids with the same features: containing surfactants/co-surfactants, solidness at various temperatures and low melting points. As for lipids cetyl palmitate, Compritol^®^ 888 ATO, Precirol^®^ ATO5, glycerol monostearate, stearic acid, stearyl alcohol, and for surfactants (function also as a stabilizer) dimethyl dioctadecyl ammonium bromide and Tween^®^ 80 and poloxamer 188 have been used as the most common ones. The appropriate choice of lipids, surfactants and the composition of SLNs (the solid core: 0.1–30% w/w, surfactants: 0.5–5% w/v) influences their release profile, drug encapsulation, stability over time, surface charge, polydispersity, size and physicochemical features.

 SLNs have several advantages; (a) ability to effectively deliver both hydrophilic and lipophilic drugs to various tissues, (b) potential encapsulation with a wide range of therapeutic molecules, such as oligonucleotides, peptides, genes and other tiny nanoparticles like superparamagnetic iron oxide particles, (c) ability to protect the loaded therapeutic molecule from reticuloendothelial system clearance, (d) poor water solubility that favors the encapsulated substance for controlled and sustained release, (e) long-term stability and lower toxicity making them applicable for long-term administration, (f) due to their biocompatibility, they are easily sterilized and there is no need for organic solvents use which might influence the toxicity of the final product, (g) they have large-scale industrial production capacity, (h) with modified targeting features they can specifically target the affected tissue.

 Their disadvantages can be addressed as the following; a) encapsulated therapeutic particles export, (b) gelation predisposition, and (c) low encapsulation efficiency.^58,59^ The latter is the result of the crystallization process which leaves the lipid core internal structure without enough space for therapeutic substance loading.

## Nanostructured lipid carriers

 NLCs were introduced by Müller et al^60^ to improve the low-encapsulation efficiency of SLNs, with increased internal free space in solid lipid core structures. To synthesize such structure, a mixture of liquid and solid lipids with mono-, di- and triglycerides of different chain lengths is employed.^61^ Besides improving the encapsulation efficiency, other drawbacks of SLNS were improved, namely as stability and no drug expulsion during storage.^61,62^

 It has been proved that hydrophobic drugs have higher dissolution rate in the liquid lipids than in solid ones, leading to increased encapsulation efficiency and higher solubility of drugs which in turn even results in higher encapsulation efficiencies.^63^ However, for hydrophilic drugs a lipid conjugation approach is used, by which the functional group of the drug (e.g. amine group) is conjugated with the functional group (e.g. carboxylic acid group) of lipids like oleic acid, through carbodiimide or another type of chemistry.

 Among all the liquid lipids in use for NLCs Capmul^®^ MCM C8, L-phosphatidyl choline (PC), Tegosoft^®^ M, Tegosoft^®^ P, soy lecithin, sesame oil, Speziol^®^ EOL NF, Mygliol^®^ 812 N, almond oil, olive oil, Suppocire^®^ NC, cetiol, peanut oil, corn oil, soybean oil and oleic acid can be named. As for the surfactants N-[1-(2,3-dioleyloxy) propyl]-N,N,N-trimethyl-ammonium chloride (DOTMA), Tween^®^ 20, Tween^®^ 80, Lutrol F68, Tego Care 450, Pluronic^®^ F68, Speziol^®^ TPGS Pharma, Myrj^TM^ 59, Span^®^ 85, Eumulgin SML, Cremophor^®^ RH, Cremophor^®^ EL. For NLCs synthesis the following ratio could be employed: the surfactant concentration 0.25–6% w/v, solid and liquid lipid 4:1–1:4, total lipid concentration 1–30% w/v.

## Novel drug delivery strategies for brain tumors

###  Targeting moieties

 The inability to locate or target cancer tissue; deposition of drug in the wrong tissue; Adsorption of drug by intact cells; Low in-site amount of therapeutic nanoparticles following poor cell endocytosis have been mentioned as the main reasons of failure in cancer targeted drug delivery of new biotechnological medicines.

 Hence, exploiting cell and molecular biology techniques, specific targeting moieties for GBM have been discovered which have been under investigation for therapeutic purposes by nanotechnology. There are two ways to target tumor cells by nanoparticles; passive in which the tiny therapeutic molecules of drug reach and accumulate in cancer tissue by taking advantage of extravasation process through the “leaky” vessels, and active in which specific ligands attach to a specific receptor or molecular marker of the cancer cell which this feature in turn can be used for targeted-drug delivery.^64^ Since then, a new concept called ”biosensors” has been emerged in nanotechnology and drug delivery systems,^65^ and there have been clinical trials to investigate this concept for active drug delivery (Table 2).

**Table 2:** Preclinical and clinical trials of nanomedicines for the treatment of brain tumors (http://www.clinicaltrials.gov)

**Clinicaltrials ID**	**Condition**	**Intervention**	**Phase**
NCT00734682	Glioblastoma, gliosarcoma, anaplastic astrocytoma, anaplastic oligodendroglioma	Drug: Nanoliposomal CPT- 11	Phase 1
NCT02340156	Recurrent glioblastoma	Genetic: SGT-53 Drug: Temozolomide	Phase 2
NCT00769093	Brain neoplasms	Drug: Ferumoxytol	Phase 1
NCT00313599	CNS tumor	Drug: lapatinib, paclitaxel	Phase 1
NCT01967810	Glioma glioblastoma brain tumor, recurrent	Drug: ANG1005 Drug: Bevacizumab	Phase 2/ ongoing
NCT02048059	Breast cancer brain metastases	Drug: ANG1005	Phase 2/ ongoing
NCT02820454	Brain metastases	Drug: AGuIX Radiation: whole brain radiation therapy	Phase 1/ongoing
NCT03020017	Gliosarcoma, recurrent GBM	NU-0129, spherical nucleic acid (SNA) arranged on the surface of a small spherical gold nanoparticle/ targeted molecular therapy	Early Phase 1/on going
NCT02766699	GBM, astrocytoma, grade IV	EGFR(V)-EDV-Dox [EGFR (vectibix sequence) targeted EnGeneIC dream vectors containing doxorubicin]	Phase 1/ongoing
NCT03086616	Diffuse intrinsic pontine glioma	CED of nanoliposomal irinotecan (nal-IRI)	Phase 1/ongoing
NCT02022644	High grade glioma	CED of nanoliposomal irinotecan	Phase 1/ongoing
NCT01386580	Brain metastases, malignant glioma	2B3-101 (phase1) /Trastuzumab (phase2)	Phase1&2/completed (2014)
NCT02861222	Malignant glioma	Liposomal doxorubicin (MYOCET)	Phase 1/completed (2013)
NCT00944801	GBM	PEGylated liposomal doxorubicin	Phase1&2/completed (2009)
NCT00019630	Brain tumor	Doxorubicin HCl liposome	Phase 1/completed
NCT01906385	GBM, astrocytoma	Rhenium nanoliposome	Phase 1&2/ongoing
NCT01517464	Neoplasm	Genetic: SGT-94 Liposome	Phase 1/ongoing
NCT01266096	Malignant brain tumors	Drug: PET scan with 124I- cRGDY-PEG-dots silica nanoparticles	Phase 0/ongoing
NCT03020017	Gliosarcoma recurrent glioblastoma	Laboratory Biomarker Analysis Pharmacological Study Drug: Targeted Molecular Therapy gold nanoparticles	Early Phase1/ongoing

 Cancer cells overexpress numerous surface receptors which can be targeted by various biological ligand and moieties in drug delivery for brain cancer treatment; so far protein, peptides and antibodies have been under investigation. According to some studies (Table 3), there are various endothelial cell receptors in the brain for which there are specific ligands that can be exploited for the drug delivery purposes through the BBB. Nevertheless, given their ubiquitous expression in body cells, the risk of non-specific adverse effects might limit this approach.

**Table 3:** Targeting moieties for brain drug delivery of nanomedicines

**Class of ligand**	**Model drug**	**Receptor**	**Nanoparticle**	**Reference**
Antibodies	Loperamide	Transferrin/anti-transferrin receptor monoclonal antibodies (OX26 or R17217)	human serum albumin (NHS-PEG-MAL-5000 linker)	^66^
Antibodies	_	Transferrin	Transferrin/bovine serum albumin	^67^
Antibodies	Loperamide	Insulin/anti-insulin receptor monoclonal antibody (29B4)	human serum albumin (NHS-PEGMAL-5000 linker)	^68^
Antibodies	Anti-IL13αR mAB	IL3αR2	_	^69,70^
Antibodies	Specific mAB	CD133	Carbon Nanotubes	^71^
Peptides	paclitaxel	Angiopep (Thr-Phe-Phe-Tyr- Gly-Gly-Ser-Arg-Gly-Lys-Arg- Asn-Asn-Phe-Lys-Thr-Glu-Glu-Tyr)/ low density lipoprotein	Angiopep-conjugated poly(ethylene glycol)-co- poly(ε-caprolactone) copolymer	^72^
Peptides	loperamide	H-2N-Gly-l-Phe-d-Thr-Gly-l-Phe-l-Leu-l-Ser-O-ß-d- glucose-CONH2	Gly-l-Phe-d-Thr-Gly-l-Phe-l-Leu-l-Ser(O-β-d-glucose)- CONH2 bound to poly(D,L- lactide-co-glycolide)	^73^
Peptides	Synthetic RGD	αvβ3 integerin	_	^74,75^
Peptides	Tat protein (from HIV)	_	liposomes	^76^
Proteins	siRNA	Transferrin	Cyclodextrin polymer-based	^77^
Proteins	Chlorotoxin (from scorpion)	Membrane bound matrix metalloproteinase-2 (MMP-2)	-	^78^

## Intranasal drug delivery; a “shortcut” to brain

 Intranasal delivery is a novel therapeutic drug delivery directly to the brain through the epithelium of the olfactory nerve (cranial nerve І) and trigeminal nerve (cranial nerve V) as anatomic connections. Since the nanoparticles are administered in nasal cavity, they protect the chemotherapeutic drug from the biophysical barriers namely as BBB. However, the administered drug is not able to recognize the intact and cancer brain tissue.^79^ There have been studies of successful brain delivery of anticancer drugs (such as methotrexate, 5-fluorouracil and raltitrexed) using intranasal delivery, evaluating the cellular mechanisms, involved cellular receptor and main vectors.^80^ Given its novelty, still further studies are required to assess the benefits and drawback of this route for brain cancer treatment.

## Conclusion

 Despite promising progress in recognizing pathophysiological and cellular behavior of malignant brain tumors such as GBM, yet they remain a medical challenge with high mortality rate. Recent in-depth understanding of the biochemistry, specific markers, ligands and receptors involved resulted in novel chemotherapeutic drugs, which are mostly hydrophobic and consequently reach into insufficiently to the tumor tissue to exert their maximum therapeutic effect. Furthermore, unique structure of BBB and BBTB which function as a barrier prevent in-site anticancer drug delivery to the cancer tissue. Hence, various nanomedicines have been investigated with in-vitro/in-vivo studies to overcome these barriers to provide high drug diffusion, controlled drug release profile, tumor-specific targeting and long-term blood circulation. Lipid nanoparticles among all offer promising drug delivery and can be modified in such a way to by-pass barriers and deliver the encapsulated therapeutics to the affected brain tissue. They also offer so many advantages over other polymeric nanoparticles with enhanced efficacy, reduced toxicity and enhanced drug stability.

## Competing Interests

 The author declares no conflict of interest.

## Ethical Approval

 There is none to be disclosed.
